# Systematic review of patient-specific pre-operative predictors of pain improvement to endometriosis surgery

**DOI:** 10.1530/RAF-20-0057

**Published:** 2021-03-03

**Authors:** Elizabeth Ball, Babu Karavadra, Bethany Jade Kremer-Yeatman, Connor Mustard, Kim May Lee, Sharandeep Bhogal, Julie Dodds, Andrew W Horne, John Allotey, Carol Rivas

**Affiliations:** 1 Department of Obstetrics and Gynaecology, The Royal London Hospital, Barts Health NHS Trust, London, UK; 2 Women’s Health Research Unit, Queen Mary University of London, London, UK; 3 Department of Gynecology, Norfolk & Norwich University Hospital, Norwich, UK; 4 Poole Hospital NHS Foundation Trust, UK; 5 Barts and the London Pragmatic Clinical Trials Unit, Queen Mary University of London, London, UK; 6 Women’s Health Research Unit, Barts and the London School of Medicine and Dentistry, Queen Mary University of London, London, UK; 7 MRC Centre for Reproductive Health, University of Edinburgh, UK; 8 Institute of Metabolism and Systems Research and Institute of Applied Health Research, University of Birmingham, Birmingham, UK; 9 UCL Social Research Institute, University College London, London, UK

**Keywords:** endometriosis, laparoscopy, systematic review, surgery

## Abstract

**Background:**

Up to 28% of endometriosis patients do not get pain relief from therapeutic laparoscopy but this subgroup is not defined.

**Objectives:**

To identify any prognostic patient-specific factors (such as but not limited to patients’ type or location of endometriosis, sociodemographics and lifestyle) associated with a clinically meaningful reduction in post-surgical pain response to operative laparoscopic surgery for endometriosis.

**Search strategy:**

PubMed, Cochrane and Embase databases were searched from inception to 19 May 2020 without language restrictions. Backward and forward citation tracking was used.

**Selection criteria, data collection and analysis:**

Cohort studies reporting prognostic factors, along with scores for domains of pain associated with endometriosis before and after surgery, were included. Studies that compared surgeries, or laboratory tests, or outcomes without stratification were excluded. Results were synthesised but variation in study designs and inconsistency of outcome reporting precluded us from doing a meta-analysis.

**Main results:**

Five studies were included. Quality assessment using the Newcastle–Ottawa scale graded three studies as high, one as moderate and one as having a low risk of bias. Four of five included studies separately reported that a relationship exists between more severe endometriosis and stronger pain relief from laparoscopic surgery.

**Conclusion:**

Currently, there are few studies of appropriate quality to answer the research question. We recommend future studies report core outcome sets to enable meta-analysis.

**Lay summary:**

Endometriosis is a painful condition caused by displaced cells from the lining of the womb, causing inflammation and scarring inside the body. It affects 6–10% of women and there is no permanent cure. Medical and laparoscopic surgical treatments are available, but about 28% of patients do not get the hoped-for pain relief after surgery. Currently, there is no way of predicting who gets better and who does not. We systematically searched the world literature to establish who may get better, in order to improve counselling when women choose treatment options. We identified five studies of variable quality showing: More complex disease (in specialist hands) responds better to surgery than less, but more studies needed.

## Introduction

Endometriosis is a chronic inflammatory condition affecting 6–10% of women of reproductive age, defined by the presence of endometrial-like tissue outside the uterus, commonly affecting the peritoneum, ovaries and other pelvic organs (Viganò *et al.* 2004).

Endometriosis impacts on many aspects of daily life and is associated with considerable costs to health services and society (Simoens *et al.* 2012). Commonly, women with endometriosis experience infertility and fatigue as well as pain, the latter often worsening during menses (dysmenorrhoea) and sexual intercourse (dyspareunia). In addition, pain may occur during bowel movements (dyschezia) or in a non-cyclical fashion.

There is no cure for endometriosis and current established treatments show an inconsistent response. Laparoscopic removal of endometriosis (therapeutic laparoscopy) remains the mainstay of treatment for endometriosis-associated pain (as described above) as a stand-alone intervention (Zanelotti & Decherney 2017), after the failure of or in conjunction with medical treatment (Duffy *et al.* 2014).

There is a distinction between diagnostic and therapeutic laparoscopies, and clinicians are advised to use a combined ‘see and treat’ approach for most cases (Ball *et al.* 2008). A recent meta-analysis (Leonardi *et al.* 2020), which included two studies also reviewed in this paper (Sutton *et al.* 1994, Abbott, 2004 #12), demonstrated that operative laparoscopy was more effective for pain relief at 6 months than diagnostic laparoscopy (*n* = 102; RR 2.65; 95% CI 1.61–4.34, *P* < 0.001).

Unfortunately, between 20% (Abbott *et al.* 2004) and 28% (Sutton *et al.* 1994) of women with endometriosis pain do not respond to therapeutic laparoscopy (pre- and post-operative pain scores are not different), but it is not known which subgroup of women will respond and which will not. A recent meta-analysis (Leonardi *et al.* 2020) entitled ‘When to Do Surgery and When Not to Do Surgery for Endometriosis’ failed to identify sufficient evidence to answer this question.

The location and the severity of endometriosis commonly staged 1–4 using the revised American Fertility Society grading system (r-AFS) (The American Fertility Society 1985) may correlate with patients’ symptoms (Fauconnier *et al.* 2002, Sinaii *et al.* 2008) and it could be hypothesized that these factors may also have prognostic value for treatment response.

If clinicians knew which subgroup of endometriosis patients benefitted from laparoscopic surgery, they would be better able to counsel their patients and manage their expectations. Access to therapeutic laparoscopy, which is a costly, limited resource associated with anaesthetic and surgical risks, could be better managed.

This review aims to determine which women will benefit from therapeutic laparoscopy for endometriosis.

## Methods

A systematic review was performed using a prospectively registered protocol as part of a more extensive investigation (PROSPERO CRD42018108604, 04 September 2018) within the CRESCENDO project (peer and Patient and Public Involvement (PPI)-reviewed, NIHR PB-PG-0317-20018). Findings are reported in line with PRISMA guidelines. The search was performed on PubMed, Cochrane and Embase databases from inception to 19 May 2020 without language restrictions. At the time of protocol writing, no relevant core outcomes were published, though one has since been developed (Duffy *et al.* 2020). In the absence of predictor variables associated with a favourable surgical outcome published in reviews or guidelines, we chose an inclusive search strategy. The search is detailed in Supplementary material (see section on supplementary materials given at the end of this article). A manual search of reference lists of included articles, as well as backward and forward citation tracking, supplemented the database search. When clarification on data was required, authors were also contacted.

Two reviewers (E B and B K) screened titles and abstracts separately for eligible articles and reviewed the full-texts of these articles for final study selection. Disagreements were resolved by discussion between reviewers and with a third reviewer (J A).

Our interest was in prognostic factors that can be used to identify women most likely to experience pain relief from laparoscopic surgery for the treatment of endometriosis-related pain. Only patient-specific pre-operative factors were explored, surgery-specific factors were beyond the remit of this review, as the former would be the most relevant for patient counselling before surgery. Thus, the inclusion criteria, using the PECO format (Morgan *et al.* 2018), were:

Patients: women with endometriosis
Exposure: Women, for whom the presence of any type of prognostic patient-specific factor was reported (this could be any sociodemographic, lifestyle and disease-related factors). We did not specify the prognostic factor before a priori, but approached the search with an open mind and recorded the prognostic factors that were available in the literature and where the pain outcomes were stratified by those predictors.
Comparison: Women without the prognostic factor of interest (e.g. parous women (exposure) nulliparous women (non-exposure))
Outcomes: Improved dysmenorrhoea, dyspareunia, non-cyclical pelvic pain and dyschezia or global pain reported after at least 6 months on the visual analogue score (VAS) or as ‘better’ or ‘improved’ vs ‘not better’ or ‘not improved’

Pain relief after surgery had to be reported stratified by the prognostic factor, to allow, if data were available, for the construction of a 4 × 4 table. This means that studies without a comparative element were not included.

Excluded from teh study were recurrence and re-operation rates as measures for surgical outcomes, fertility outcomes, a post-operative follow-up time of less than 6 months (the minimum the research group agreed necessary to judge genuine surgical outcomes), studies comparing different surgical techniques, or laboratory tests as predictor variables, reports without predictor variables, abstracts, case reports, conference proceedings, and review articles.

E B and C M independently extracted the data on pre- and post-operative pain scores stratified by risk factors. E B and C R assessed the quality of studies using the Newcastle–Ottawa scale (Wells *et al.* 2019).

Findings were reported as a qualitative synthesis due to a paucity of data and variation in reporting, which precluded meta-analysis.

## Results

### Search results and risk of bias

**Figure 1:**
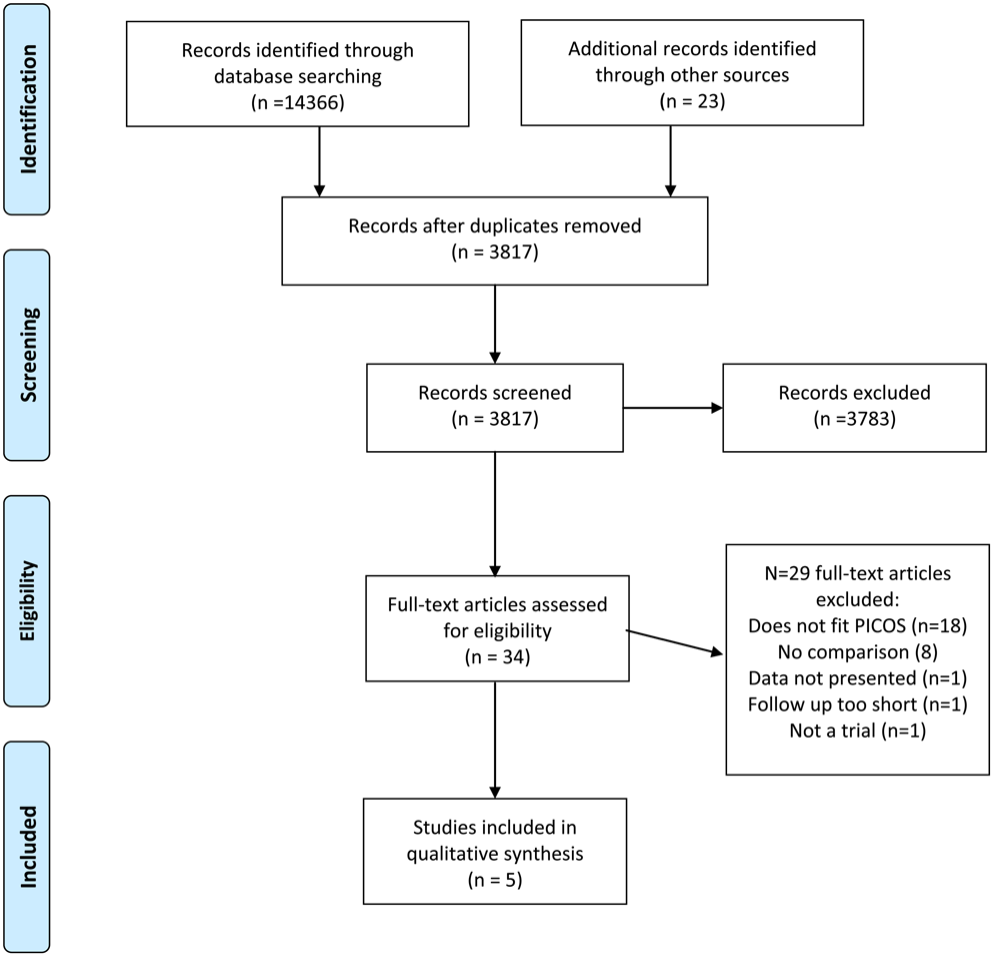
PRISMA flow diagram.

The search returned 14,366 citations; additional backward and forward citation tracking returned one additional paper. After the removal of duplicates, 34 full-text papers were obtained. Of these, 29 were excluded after inclusion and exclusion criteria were applied (Fig. 1 and Supplementary Table 1). We included five studies (*n* = 606) (Abbott *et al.* 2003, Chopin *et al.* 2005, Banerjee *et al.* 2006, Milingos *et al.* 2006, Ghai *et al.* 2020), two retrospective (Chopin *et al.* 2005, Ghai *et al.* 2020), three prospective. All were from specialist clinics from the global north and included all endometriosis stages (study details Table 1). The results reported are presented in Table 2 and the findings of the included studies are detailed in Table 3.

**Table 1:** Characterisation of studies.

Reference	Design	Cohort studied	Average age (years)	Parity	Setting	Study size and attrition			EM location	EM stages	Prev. treatment	Operative approach	Hist. confirm.	Follow-up time from baseline, y	Pre- and post-operative outcome measures
						LAP^‡^	EM	Follow-up							
Abbott *et al.* (2003)	POC	ES 1–4	31 (20–48)	0: 80/175 (60%)	2 UK UH	261	176	135	OE: 38%; USL 88%	Stage 1: 28%; Stage 2: 28%; Stage 3: 17%; Stage 4: 41%	Analgesia: 70%; HT: 70% Prev. LAP: 70%	CLET: 100%	Yes	5 (3.2 (2–5)^⁑^)	VAS (10 cm) presented as median and IQR for dysmenorrhoea, dyspareunia, non-menstrual PP, dyschezia)
Chopin *et al.* (2005)	ROC	DE^†^	31.7 ± 5.4	0.3 ± 0.61 (0–3)	1 French UAH	241	241	132	USL: 59.9%; vagina: 18.9%; bladder: 9.8%; intenstine: 12.1%; multiple locations: 39.4%	Stage 1: 20.5%; Stage 2: 34.1%; Stage 3: 24.2%; Stage 4: 21.2%	HT: 56%; Prev. LAP: 0.9 ± 1	CLET: 87.1%; LT: 12.9%	Yes	3.7 ± 2.0 (median: 3.3)	VAS (10 cm) for each pain type (dysmenorrhoea, dyspareunia, CPP, dyschezia, lower urinary tract symptoms)
Banerjee *et al.* (2006)	POC	ES 1–4 and no EM	NS		1 UK tertiary EM centre in DGH	108	88	46	OE: 14%; USL: 59%; recto-vaginal: 43%; pouch of Douglas: 43%; intestine: 47%	Stage 1: 39%; Stage 2: 16%; Stage 3: 9%; Stage 4: 36%	NS	CLET: 100%	Yes	18 months post-surgery	Pre- and post-operative global pain scores; these are a sum of VAS (0-5) for dysmenorrhoea, dyspareunia, non-cyclical pelvic pain, menstrual dyschezia, non-menstrual dyschezia, menstrual backache, non-menstrual backache
Milingos *et al.* (2006)	POC	ES 1–4	NS		1Greek UH	258	101	95	OE:61.8%; USL: 59%; recto-vaginal septum: 10.3%	Minimal and mild: 21.4%; moderate and severe: 71.6%	Prev. LAP: 14.7%	CLET; LT: 6.3%	no	6 months post-surgery	VAS (10) for each pain type dysmenorrhoea, dyspareunia, non- menstrual pelvic pain. Pain scores grouped as 0, 1-5, 6-7, 8-10
Ghai *et al.* (2020)	RS*	ES 1-4	NS		1 UK tertiary EM centre in DGH	102	96	100	ND	Stages 1–3 ‘superficial’: 48%; stage 4 with bowel involvement ‘severe’: 52%	GRA: 6/12	Stages 1–3: laser destruction or excision; Stage 4: excision (97.1 % CLEP); LT: 2.9%	Severe: yes; superficial: NS	12 months post-surgery	Pre- and post-operative pain component measured with EPH30 questionnaire

*Analysis of data from previous databases (1, 2); ^†^with infiltration of USL or Bladder/intestine/vagina; ^‡^women with CPP who had laparoscopy; ^⁑^mean (range).

CLET, complete laparoscopic excisional treatment; DGH, district general hospital; EM, endometriosis; ES, endometrial stage; GRA; gonadotropin receptor antagonist; Hist. confirm., histological confirmation; HT, hormonal treatment; LAP, laparoscopy; LT, laparotomy; ND, not detailed; NS. Not stated; OE, ovarian endometrioma; POC, prospective observational cohort; Prev. previous; ROC, retrospective observational cohort; RS, retrospective secondary; UAH, university affiliated hospital; UH, university hospital.

**Table 2:** Presentation of results.

Reference	Outcomes stratified by risk factors	Presented as	Authors’ conclusions
Abbott *et al.* (2003)	Outcomes of dysmenorrhoea, dyspareunia, non-menstrual PP, dyschezia pre and postop scores stratified by endometriosis AFS staging 1–4	Median, IQR and *P*	The results from sub-analysis examining pain scores by stage suggested a reduction in pain for all four parameters examined.
	QOL measures reported but not stratified		
Chopin *et al.* (2005)	Outcomes of dysmenorrhoea, dyspareunia, CPP, Dyschezia, lower Urinary tract symptoms pre and postop scores stratified buy anatomical location (USL, Vagina, bladder, intestine)	Mean, s.d. and *P*	The results presented show that for each location in the surgical classification, the mean scores for the five symptoms according to the numerical rating scale were significantly lower postoperatively. This result is nearly significant when the group-specific sample sizes of patients are very small.
Banerjee *et al.* (2006)	Global pain score stratified by no endometriosis, only superficial endometriosis, deep ± superficial endometriosis	Mean pain score and s.d.	This small study suggests that surgical therapy does not reduce pain scores in superficial endometriosis but is valuable in the treatment of deep or infiltrating disease.
Milingos *et al.* (2006)	Outcomes for dysmenorrhoea, deep dyspareunia and non-menstrual pain stratified by: 1. number of improved patients (reduction ≥2 cm VAS was considered significant); 2. change in pain scores (graph) for severity AFS score <16 (group 1) and 16+ (group2)	Graphics: changes in pain scores with *P*; proportion improved patients and *P*	Cases with advanced disease seem to benefit the most
Ghai *et al.* (2020)	Outcome is any reduction of EPH 30 pain score reduction (responders): 1. comparison between the proportion of responders among women with severe and superficial endometriosis; 2. stratification within the superficial and severe groups by anxiety and depression HADS scores, feeling of control, emotional wellbeing, sexual relationship and pain EPH30 scores, VAS for dysmenorrhoea, dyspareunia, CPP, dyschezia	proportion of non-responders in severe and superficial endometriosis with *P* value median and range for HADS, EPH30 domains and for VAS with *P* value for superficial and severe endometriosis	Severity of disease and pain and pain may be used to predict response to surgery

Considering the risk of bias for the five studies, one scored low (Chopin *et al.* 2005), one medium (Ghai *et al.* 2020), and four (Abbott *et al.* 2003, Banerjee *et al.* 2006, Milingos *et al.* 2006, Ghai *et al.* 2020) high using the Newcastle–Ottawa tool. While scoring highly in other domains, three studies scored low on ‘comparability’ which may be the result of poor reporting rather than poor study design.

We found reports which stratified post-surgical pain relief by disease severity and anatomical site. There were no reported data on the predictive role of sociodemographic factors (for instance age and parity).

### Study participants

Chopin *et al.* (2005) retrospectively reported data from a continuous series of women (age not stated) from a French university-affiliated hospital who reported pain (dysmenorrhoea, deep dyspareunia, chronic pelvic pain (CPP) or a pain combination) and deep endometriosis (DE) affecting at least a uterosacral ligament (USL). Of the 241 recruited women with laparoscopy-proven DE, 132 were included with complete follow-up. Only women with a histological lesion of ≥5 mm depth were included.

Banerjee *et al.* (2006) recruited women (age not stated), with symptoms suggestive of endometriosis from CPP clinics in a district hospital with tertiary level endometriosis care. One hundred and eight women were recruited; 88 women had histologically confirmed endometriosis, two women had no endometriosis. Of the 88 with endometriosis, 44 women had complete datasets and were analysed.

Milingos *et al.* (2006) recruited 274 women with CPP of ≥6 months from university fertility and laparoscopy clinics, of whom 258 underwent laparoscopy, excluding women with a pouch of Douglas obliteration or requiring hysterectomy. One hundred and one women were visually diagnosed with endometriosis during laparoscopy.

Abbott *et al.* (2003) reported 254 women referred with pain symptoms suggestive of endometriosis to two specialist units of whom 132 were included in the analysis. The mean age was 31 years (20–48), 6% were nulliparous, 70% had required analgesia for pain and 73% had hormonal treatment. Seventy per cent had at least one prior diagnostic or operative laparoscopy.

Ghai *et al.* (2020) reported a secondary analysis of existing databases (Kent *et al.* 2014, 2016) of198 women who had endometriosis surgery. In the group with severe endometriosis, 2.9% were converted to laparotomy. The authors do not report demographics but state no difference between responders and non-responders in age and stage of endometriosis within superficial and deep endometriosis (Ghai *et al.* 2020). Three studies recruited in England (Abbott *et al.* 2003, Banerjee *et al.* 2006, Ghai *et al.* 2020) one in Greece (Milingos *et al.* 2006) and one in France (Chopin *et al.* 2005).

Authors state CPP as an indicator for surgery; one study also includes fertility (Ghai *et al.* 2020). In studies that stated the data (Abbott *et al.* 2003, Chopin *et al.* 2005), women averaged 31 years and a large proportion were childless (Table 1). Ethnicity and other sociodemographic factors were not reported. All studies reported dropouts (Table 1). In one study (Banerjee *et al.* 2006), this involved half of the women who had laparoscopically confirmed endometriosis. Apart from Banerjee *et al.* (2006), all other authors listed previous surgical and medical treatments (Table 1).

In all studies, the aim was for complete laparoscopic endometriosis removal. The surgical approach depended on the depth and location. Milingos *et al.* (2006) described ablation of implants (not further specified), lysis of adhesions and excision of fibrosis/endometrioma. Six cases of ‘frozen pelvis’ were converted to open hysterectomy with bilateral oophorectomy and were excluded. Banerjee *et al.* (2006) described excision with monopolar diathermy; rectovaginal and bilateral USL lesions were removed en-bloc, ovarian endometrioma drained and excised, vaginal and bladder endometriosis fully excised and bowel endometriosis treated with shaving or disc resection. Chopin *et al.* (2005) described a ‘see and treat approach’ and excision of all endometriosis lesions ± ureterolysis. Endometrioma were excised; superficial implants were coagulated. Bladder and USL lesions were excised, and vaginal endometriosis was treated with laparoscopically assisted resection. Intestinal lesions were treated by laparoscopy or laparotomy (*n* = 16 not further specified). Abbott *et al.* (2003) reported a previously published excisional technique without hormonal pre-treatment (Garry *et al.* 2000). Ghai *et al.* (2020) described laser ablationor ultrasonic excision of superficial endometriosis. All DE patients had bowel involvement treated with bowel shaving, disc excision or anterior resection. Women received six months of reoperative gonadotropin-releasing hormone antagonist.

In two studies (Chopin *et al.* 2005, Milingos *et al.* 2006), a proportion of complex cases were either converted to or planned as laparotomies. Milingos *et al.* (2006) excluded six cases due to conversion to open surgery.

Apart from Milingos *et al.* (2006) and Ghai *et al.* (2020), who included ablation of endometriosis, all others report histological confirmation. The duration of follow up ranged from 6 months (Milingos *et al.* 2006) to 9 years (Abbott *et al.* 2003). Three studies scheduled follow up at a single timepoint: Milingos *et al.* (2006) 6, Ghai *et al.* (2020) 12 and Banerjee *et al*. (2006), at 18 months. Chopin *et al.* (2005) reported a mean follow-up of 3.3 years (range 1.0–9.1) and Abbott *et al.* (2003) a meanfollow-up of 3.7 years (range 2–5). Follow-up rates were 94% (Banerjee *et al.* 2006), 76% (Abbott *et al.* 2003), 72% (Ghai *et al.* 2020) for severe endometriosis, 54% (Chopin *et al.* 2005) and 52% (Milingos *et al.* 2006).

Dysmenorrhoea, dyspareunia, and also non-menstrual pelvic pain or CPP (used synonymously) were measured in all studies. Additional symptoms were menstrual and non-menstrual dyschezia, menstrual and non-menstrual backache and lower urinary tract symptoms. Apart from Ghai *et al.* (2020) to Banerjee *et al.* (2006), researchersused the 10 cm VAS for pain. Banerjee used a 0–5 cm VAS for pain and calculated one global score for each participant. Milingos *et al.* (2006) grouped the VAS results measured as 0 cm, 1–5 cm, 6–7 cm and 8–10 cm, when testing the correlation between pain and endometriosis severity. Furthermore, they created the binary measurement ‘improved’ vs ‘non-improved’ (reduction of ≥2 points) when comparing the post-operative pain reduction of minimal/mild with moderate/severe endometriosis. Ghai *et al.* (2020) measured pain changes using the EPH-30 questionnaire, defining any pain decrease as improvement.

### Endometriosis severity

Regarding endometriosis severity (Table 1), all studies included all endometriosis stages. The proportion of moderate and severe endometriosis combined is highest in the Milingos study (71.6%) (Milingos *et al.* 2006), followed by the Abbott study (58%) (Abbott *et al.* 2003) and in the Chopin (Chopin *et al.* 2005) and Banerjee Banerjee *et al.* (2006) studies, who both report 45%. Ghai *et al.* (2020) reports DE (excluding moderate severity endometriosis) at 48%. The high proportion of severe endometriosis is likely due to recruitment from specialised centres. The two predictor variables by which outcomes were stratified were endometriosis severity and anatomical site. Within the group of severe endometriosis, Ghai *et al.* (2020) reported higher pre-operative pain and lower feeling of control scores associated with response to surgery. Study findings are shown in Table 3.

**Table 3:** Study findings.

Reference	Findings					
	Condition	Result				
		Scores*	*P*-values	Preop^†^	Postop^†^	Delta
Abbott *et al*. (2003)						
	Dysmenorrhoea					
	All stages	9 vs 3.3	<0.0001			
	Stage I EM	8 vs 2	<0.0001			
	Stage II EM	8 vs 4.5	<0.0001			
	Stage III EM	9 vs 3.5	<0.0001			
	Stage IV EM	9 vs 2	<0.0001			
	Non-menstrual pelvic pain					
	All stages	8 vs 3	<0.0001			
	Stage I EM	6 vs 3	0.036			
	Stage II EM	6 vs 3.3	<0.0001			
	Stage III EM	6 vs 2.9	0.046			
	Stage IV EM	7 vs 2.4	<0.0001			
	Dyspareunia					
	All stages	7 vs 0	<0.0001			
	Stage I EM	7 vs 2.6	0.002			
	Stage II EM	5.5 vs 1.7	0.005			
	Stage III EM	6 vs 0	0.004			
	Stage IV EM	6 vs 0	<0.0001			
	Dyschezia					
	All stages	7 vs 2	<0.0001			
	Stage I EM	6 vs 3.1	0.035			
	Stage II EM	6 vs 2.7	0.006			
	Stage III EM	4 vs 0	0.12			
	Stage IV EM	5 vs 2	0.002			
Chopin *et al.* (2005)						
	USL (*n* = 78)					
	Dysmenorrhea (*n* = 68)		0.0001	7.68 ± 2.08 (0–10)	3.31 ± 3.31 (0–10)	4.36 ± 3.61
	Deep dyspareunia (*n* = 61)		0.0001	6.41 ± 2.47 (0–10)	2.12 ± 2.71 (0–10)	4.30 ± 3.29
	Dyschezia (*n* = 39)		0.0001	6.44 ± 2.59 (0–10)	2.72 ± 3.12 (0–10)	3.72 ± 4.00
	LUTS (*n* = 21)		0.0011	5.52 ± 0.69 (2–8)	2.29 ± 3.23 (0–8)	3.24 ± 3.02
	CPP (*n* = 36)		0.0001	7.36 ± 1.46 (3–10)	3.25 ± 3.83 (0–10)	4.11 ± 3.34
	Vagina (*n* = 25)					
	Dysmenorrhea (*n* = 23)		0.0001	8.00 ± 1.48 (5–10)	2.82 ± 3.33 (0–9)	5.17 ± 3.70
	Deep dyspareunia (*n* = 21)		0.0001	6.77 ± 1.73 (4–10)	1.62 ± 3.03 (0–9)	5.14 ± 2.97
	Dyschezia (*n* = 17)		0.0007	6.77 ± 2.17 (4–10)	2.35 ± 3.10 (0–8)	4.41 ± 3.20
	LUTS (*n* = 4)		0.0679	4.50 ± 1.73 (3–7)	0.00 ± 0.00 (0–0)	4.50 ± 1.73
	CPP (*n* = 8)		0.0171	7.63 ± 1.60 (5–10)	1.62 ± 3.11 (0–9)	6.00 ± 3.25
	Bladder (*n* = 13)					
	Dysmenorrhea (*n* = 13)		0.0022	9.23 ± 1.09 (7–10)	2.23 ± 2.95 (0–7)	7.00 ± 3.27
	Deep dyspareunia (*n* = 9)		0.0117	7.56 ± 2.13 (4–10)	2.44 ± 2.60 (0–7)	5.11 ± 3.76
	Dyschezia (*n* = 4)		0.0679	7.50 ± 2.08 (5–10)	0.00 ± 0.00 (0–0)	7.50 ± 2.08
	LUTS (*n* = 12)		0.022	7.50 ± 2.24 (3–10)	0.00 ± 0.00 (0–0)	7.50 ± 2.24
	CPP (*n* = 1)			5.0 (5–5)	0.00 (0–0)	5.0
	Intestine (*n* = 16)					
	Dysmenorrhea (*n* = 16)		0.0004	9.00 ± 0.97 (8–10)	1.94 ± 2.77 (0–8)	7.06 ± 2.82
	Deep dyspareunia (*n* = 13)		0.0015	6.77 ± 2.13 (3–10)	2.08 ± 2.75 (0–9)	4.69 ± 2.32
	Dyschezia (*n* = 11)		0.0033	6.91 ± 2.55 (3–10)	1.09 ± 2.07 (0–6)	5.82 ± 2.71
	LUTS (*n* = 4)		0.0679	7.00 ± 1.83 (5–9)	1.00 ± 2.00 (0–4)	6.00 ± 3.16
	CPP (*n* = 6)		0.0277	9.17 ± 0.98 (8–10)	3.50 ± 3.89 (0–8)	5.67 ± 4.13
Banerjee *et al.* (2006)	Difference pre- to post-operative scores: 5.2 points ± 3.6 for dysmenorrhea, 4.6 points ± 3.1 for deep dyspareunia, 4.4 points ± 3.7 for painful defecation during menstruation, 4.9 ± 3.2 for LUTS during menses, and 4.6 points ± 3.4 for noncyclic chronic pelvic pain. Comparable results observed for patients in each group according to the surgical classification of their DIE lesions: USL (*n* = 78 patients); vagina (*n* = 25 patients); bladder (*n* = 13 patients); and intestine (*n* = 16 patients).					
Banerjee *et al*. (2006)	Difference pre to postoperative scores: 5.2 points ± 3.6 for dysmenorrhea, 4.6 points ± 3.1 for deep dyspareunia, 4.4 points ± 3.7 for painful defecation during menstruation, 4.9 ± 3.2 for lower urinary tract symptoms during menses, and 4.6 points ± 3.4 for noncyclic chronic pelvic pain. Comparable results observed for patients in each group according to the surgical classification of their DIE lesions: USL (*n* = 78 patients); vagina (*n* = 25 patients); bladder (*n* = 13 patients); and intestine (*n* = 16 patients).
Milingos *et al*. (2006)	Postoperatively dysmenorrhea improved in 43% of cases in group 1 (superficial endometriosis), vs 66% of cases in group 2 (deep endometriosis) (*P* = 0.0037). For deep dyspareunia, improvement was reported by 33% in group 1, vs 67% in group 2 (*P* = 0.074). Scores for Improvement in non-menstrual pain was not significantly different between the two groups (67% vs 56%). Global pain scores (s.d., pre vs post-operative) were 17.5 (7.8) vs 16.1 (6.7), *P* = 0.43 for superficial endometriosis, and 19.2 (7.2) vs 14.5 (8.9), *P* = 0.004 for deep endometriosis ± superficial (figures not given for deep alone).
Ghai *et al*. (2020)	Higher proportion of women with severe endometriosis (*n* = 86/96) than women with superficial endometriosis (77/102; *P* = 0.0089) respond to surgery. Women with severe endometriosis were more likely to respond to surgery of they have higher preoperative EPH 30 pain scores (median 66, range 24–83) as compared to lower scores (median 50; range 20.5–63.6). In this group response to surgery was associated with lower scores for ‘feeling of control’ (60.25; range 47.7–72.7 vs 62.5 vs 45.8–70.8)

*Pain scores (median VAS baseline versus follow-up 2–5 years) were all significantly reduced for conditions presented; ^†^values presented as mean ± s.d. (range).

EM, endometriosis; LUTS, lower urinary tract symptoms.

All five included studies reported endometriosis severity; four considered either AFS stages 1–4 (Abbott *et al.* 2003, Chopin *et al.* 2005) or depth of invasion (superficial/deep) in the relevant analysis (Banerjee *et al.* 2006, Ghai *et al.* 2020). Milingos *et al.* (2006) dichotomised severity into minimal/mild (AFS scores < 16) and moderate/severe endometriosis (≥16), Ghai* et al*. (2020) reported superficial (stages 1–3) vs severe disease (stage 4).

### Endometriosis-related pain and disease severity

Abbott *et al.* (2003) reported the median and interquartile ranges of the pre-operative and post-operative pain scores for different pain types and compared pain scores for endometriosis stages 1–4 before and after surgery. This study did not compare pain reduction before and after surgery between different stages of endometriosis (such as a test for trend). The reduction in dysmenorrhoea is consistently highly statistically significant (*P* < 0.001) across all endometriosis stages, but women with stage 4 endometriosis showed the highest magnitude in pain reduction across the pain types (dyspareunia <0.0001, non-menstrual pelvic pain <0.0001, dyschezia 0.002). Other stages, while still showing a significant reduction in pain showed lower levels of statistical significance. Only patients with stage 3 endometriosis showed no evidence of pain reduction from dyschezia (*P* = 0.12).

Milingos *et al.* (2006) reported higher pre-operative scores for dysmenorrhea and dyspareunia in moderate/severe (group 2) than minimal/mild endometriosis (group 1) (*P* = 0.014 and *P* < 0.0001, respectively). The authors compared changes in pain scores pre- to post-operatively in two analyses. First, ‘change in pain score’ was depicted graphically for minimal/mild and moderate/severe endometriosis. Without providing numerical data, the authors reported the magnitude for pain score reduction for dyspareunia to be higher in the group with moderate/severe endometriosis (*P* = 0.04). Differences for dysmenorrhoea and for non-menstrual pelvic pain were not statistically significant (*P* = 0.082 and *P* = 0.56, respectively). For dysmenorrhoea, the differences may have been clinically significant, as the authors reported a benefit.

Secondly, the authors looked at subgroups of women, who had ‘improved’ pain scores for dysmenorrhoea (*n* = 52), dyspareunia (*n* = 38), and non-menstrual pain (*n* = 30) after surgery (≥2 cm VAS reduction) and compared the proportions with minimal/mild and moderate/severe endometriosis. Regarding women with improved dysmenorrhea (*n* = 52), 43% had minimal/mild and 66% moderate/severe endometriosis (*P* = 0.0037). Of the women reporting improved deep dyspareunia (*n* = 38), 33% had minimal/mild and 67% had moderate/severe endometriosis (‘not significant’) and for non-menstrual pain (*n* = 30) 67% had minimal/mild and 56% had moderate/severe endometriosis (‘not significant’).

Banerjee *et al.* (2006) reported a global pain score for three groups of women: no endometriosis, isolated superficial endometriosis and DE ± superficial endometriosis. Global scoring was 35 maximum points, the sum of 0–5 points for each of dysmenorrhoea, dyspareunia, non-cyclical pelvic pain, menstrual dyschezia, non-menstrual dyschezia, menstrual backache, non-menstrual backache. The surgeon visually distinguished between superficial peritoneal and deep infiltrating/nodular lesions. Data indicate a correlation between deep/superficial classification and AFS staging (chi-square test of association: X2(3) = 25.8 *P* < 0.001). Pre- and post-operative global pain scores were compared using a paired T-test in all three groups, women without endometriosis (*n* = 2; *P* = 0.30), with only superficial endometriosis (*n* = 17; *P* = 0.43), and with DE ± superficial endometriosis (*n* = 27; *P* = 0.004). The authors concluded surgery did not reduce pain scores in superficial endometriosis but was valuable in DE. We agree but note the small group size.

Ghai *et al.* (2020) reported a significantly higher proportion of women treated for severe endometriosis responding to surgery (*n* = 86/96) than for superficial disease (77/102; *P* = 0.0089). Women with severe endometriosis were more likely to respond if they had higher pre-operative EPH-30 pain scores (median: 66, range: 24–83) vs lower scores (median: 50; range: 20.5–63.6) and lower scores for ‘feeling of control’ (60.25; range: 47.7–72.7 vs 62.5, range: 45.8–70.8).

### Endometriosis-related pain and disease location

All authors, apart from Ghai *et al.* (2020), detailed endometriosis location; USL endometriosis was listed in four studies, ovarian endometrioma in three (Abbott *et al.* 2003, Banerjee *et al.* 2006, Milingos *et al.* 2006) and rectovaginal septum (Milingos *et al.* 2006) and intestinal endometriosis in two (Chopin *et al.* 2005, Banerjee *et al.* 2006).

Chopin *et al.* (2005) reported pre- and post-operative pain scores stratified by location: USL, vagina, bladder and intestine. Pre- and post-operative differences in pain scores were compared for each location, but locations were not compared with each other. Removal of USL endometriosis (*n* = 78) resulted in highly significant reduction across all five pain types (dysmenorrhoea: *P* < 0.001, deep dyspareunia: *P* < 0.001, dyschezia: *P* = 0.001, lower urinary tract symptoms: *P* = 0.011, and non-cyclical pelvic pain: *P* < 0.001). Vaginal (*n* = 25) and intestinal (*n* = 16) endometriosis excision was associated with a significant reduction of four pain types (dysmenorrhoea: *P* = 0.001 and *P* = 0.004, respectively, deep dyspareunia: *P* = 0.001 and *P* = 0.015, respectively, dyschezia: *P* = 0.007 and *P* = 0.033, respectively, and non-cyclical pelvic pain *P* = 0.022 and *P* = 0.027, respectively), but not lower urinary tract symptoms (*P* = 0.0679 and *P* = 0.0697, respectively). Removal of bladder endometriosis (*n* = 13) resulted in a significant reduction in dysmenorrhoea *P* = 0.022, deep dyspareunia *P* = 0.0117 and lower urinary tract symptoms *P* = 0.022, but not dyschezia *P* = 0.0697. Non-cyclical pelvic pain reduction could not be ascertained due to missing data.

### Excluded studies

Two excluded studies for which we obtained full texts merit further discussion. Sutton *et al.* (1994) was excluded due to limited presentation of results. Seventy-four women from gynaecology clinics with symptoms suggesting endometriosis were included. Visual assessment at laparoscopy showed minimal (*n* = 29), mild (*n* = 28) and moderate (*n* = 6) endometriosis, which was destroyed with laser and not histologically confirmed. Follow-up was 3 and 6 months post-operatively. Of the 74 recruited women 63 completed the study. Women recorded the intensity of global pain on a 10 cm VAS and also ‘how pain had changed’. The proportion of women with pain alleviation stratified by endometriosis stage was graphically displayed, without numerical values or significance testing. The proportion of women with stage 3 endometriosis is depicted at 100 ‘percentage better’ whereas the percentage in stage 1 endometriosis is depicted below 50 ‘percentage better’. The authors were unsuccessfully contacted for their raw data. However, they concluded that the severity of pain experienced by endometriosis patients may be used to predict their response to surgery.

A retrospective cohort study by Harris *et al.* (2020) recruited 972 women who underwent therapeutic laparoscopy for confirmed endometriosis. In total 398 women had complete follow-up reported 6/52 weeks post-operatively. This study was excluded because of short follow-up. Global pain was recorded as ‘pain improvement/resolution’ vs ‘no improvement’.

The proportion of women with improvement/resolution was higher if women: were ‘not Caucasian’ (*n* = 188, 67.7%) vs ‘Caucasian’ (*n* = 90, 32.4%) – OR: 0.60, CI 0.37–0.99, *P* = 0.046; were operated on by a specialised endoscopic gynaecologist (*n* = 75, 83.0%) vs not (*n* = 15, 16.7%) – OR: 0.42, CI: 0.18–0.94, *P* = 0.036; had a history of CPP (*n* = 29, 55.8%) vs not (*n* = 23, 44.2%) – OR: 2.0, CI: 1.14-3.76, *P* =0.02); had stage 3–-4endometriosis (*n* = 128, 83.1%) vs stage 1–2 (*n* = 26, 16.9%) – OR: 0.35, CI: 0.21–0.57, *P* < 0.001.

## Discussion

### Main findings

Four of the five included studies indicate that stronger pain relief after endometriosis surgery was related to more severe disease prior to surgery (Chopin *et al.* 2005, Banerjee *et al.* 2006, Milingos *et al.* 2006, Ghai *et al.* 2020). Although the current review returned a limited quantity and quality of evidence, the ‘theme severity of endometriosis’ is consistent across studies and warrants further investigation to determine whether it may be used in the future to counsel women about laparoscopic surgery for endometriosis. Endometriosis severity may be only fully understood during laparoscopy. Nonetheless, there are clinical pointers to DE, such as the severity of symptoms (Fedele *et al.* 1992, Ferrero *et al.* 2005), USL nodularity, and the ‘kissing ovary’ sign on scan, which may be used as surrogate markers for disease severity. More research is needed to quantify the value of using these in treatment decision making (Matorras *et al.* 1996, Ghezzi *et al.* 2005).

### Strengths and limitations

The strengths of this systematic review include a thorough literature review following PRISMA guidelines and assessment of studies using the Newcastle–Ottawa quality tool. However, due to the limitations of the available data and the high risk of bias scores we are unable to make definitive conclusions about predictors of surgical success.

### Interpretation

It was surprising to find so few studies focussing on patient-specific predictors of favourable surgical outcomes, given the large number of series that report evidence of a reduction of endometriosis-related pain scores after surgery (Garry *et al.* 2000, Ford *et al.* 2004, Wykes *et al.* 2006, Angioli *et al.* 2014, De la Hera-Lazaro *et al.* 2016, Byrne *et al.* 2018, Rindos *et al.* 2020) and the large numbers of affected patients.

Reviewed studies included women with advanced endometriosis, treated in specialist centres and with reported complete excision.

Surgical factors that could influence operative outcomes – such as whether excision is complete – are highly relevant to future research. Studies show less pain reduction in incomplete compared to complete surgery (Hidaka *et al.* 2012, Angioni *et al.* 2015, Cao *et al.* 2015). Thus a systematic review of three randomised controlled trials (RCTs) with 335 women indicates superior reduction of dysmenorrhea (mean difference (MD) = 0.99; 95% CI: −0.02 to 2.00; *P*  = 0.05) and dyschezia (MD = 1.31; 95% CI: 0.33–2.29; *P*  = 0.009) using excision compared to ablation, but not in dyspareunia (MD = 0.96; 95% CI: −0.07 to 1.99; *P*  = 0.07) (Pundir *et al.* 2017). Conversely, a later RCT of 73 women with endometriosis ablation and excision showed no difference in dysmenorrhoea but a difference in dyspareunia at 6 months (mean change −22.96; 95% CI: −39.06 to −6.86; *P*  = 0.01) (Riley *et al.* 2019).

The studies included in the present review used the r-AFS scoring or a score deduced from it. Whilst the AFS score was designed to predict fertility and puts strong weighting on endometriotic cysts, it may correlate less well with pain (Vercellini *et al.* 1996), whereas the ENZIAN score (Haas *et al.* 2013, Montanari *et al.* 2019) may have stronger correlation in pain in DE.

The location of endometriosis in the USL and its removal may have a special role in pain relief after surgery (Chopin *et al.* 2005, Chapron & Dubuisson 1996), and appears to be closely associated with the symptom of dyspareunia (Porpora *et al.* 1999, Fauconnier *et al.* 2002, Montanari *et al.* 2019). The presence of endometriosis is specifically associated with tenderness of the cul-de-sac or USL during examination (Yong *et al.* 2017). This can help indicate the presence of DE.

Debate remains whether surgical removal of endometriosis can relieve non-cyclical pelvic pain. Abbott *et al.* (2003), Chopin *et al.* (2005), and Banerjee *et al.* (2006), but not Milingos *et al.* (2006) reported evidence of improvement of non-cyclical pelvic pain. Pain scores for non-cyclical back pain and non-cyclical dyschezia failed to show evidence of improvement after removal of endometriosis in one paper that included these outcomes (Banerjee *et al.* 2006). These symptoms may have causes other than endometriosis, as also can non-cyclical CPP that is resistant to laparoscopic endometriosis treatment.

The use of post-operative adjuvant hormone treatment (such as the oral contraceptive pill or levonorgestrel intrauterine device) could have been a confounding variable for pain improvement, especially dysmenorrhoea. However, this detail is not provided in the studies included.

## Conclusion

The current systematic review identified severity of endometriosis as a possible predictor for surgical response based on a small number of studies, mostly assessed as having a ‘high risk of bias’. The review has also shown there is a knowledge gap that needs to be filled. A multicentre RCT to clarify if low stage endometriosis removal causes any improvement in pain scores is planned (Horne *et al.* 2019). We are also currently producing an algorithm to predict surgical success in women with confirmed or suspected endometriosis (CRESCENDO, NIHR PB-PG-0317-20018) using pre-existing databases (Daniels *et al.* 2009, Byrne *et al.* 2018, Khan 2018). Given the review findings we recommend that future studies should be designed more robustly and less heterogeneously. An important element is the reporting of pre-defined core outcome sets for endometriosis treatment (Hirsch *et al.* 2016, Duffy *et al.* 2019). With standardised reporting, studies can be adequately compared, synthesised and meta-analysed. A core outcome set for endometriosis has recently been published (Duffy *et al.* 2020) that includes overall pain, improvement in the most troublesome symptom and quality of life, and its adoption may create more substantive evidence in the future.

## Supplementary Material

Appendix S1

Table S1 Included and excluded studies

Checklist

## Declaration of interest

None of the authors declare financial, personal, political, intellectual or religious interests relating to the current paper. Andrew Horne is a Co-Editor-in-Chief of Reproduction and Fertility. Andrew Horne was not involved in the review or editorial process for this paper, on which he is listed as an author.

## Ethics approval

Given this study was a systematic review on data in the public domain it is exempt from ethics approval.

## Author contribution statement

All authors made a substantial contribution to conception and design, or acquisition of data, or analysis and interpretation of data; and in drafting the article or revising it critically for important intellectual content; and in the final approval of the version to be published. All authors agree to be accountable for all aspects of the work in ensuring that questions related to the accuracy or integrity of any part of the work are appropriately investigated and resolved. In addition, the authors carried out the following tasks: E B: conception, planning, carrying out systematic review, analysing and writing; B K: carrying out systematic review, analysing and writing; B K Y: carrying out systematic review, writing; C R: conception, planning, carrying out systematic review, analysing and writing; J A: conception, planning, carrying out systematic review, analysing and writing; C M: conception, planning, carrying out systematic review, analysing and writing; A H: conception, planning and writing; K M L: conception, planning, carrying out systematic review, analysing and writing; S B: conception, planning and writing; J D: conception and writing.
