# Supplementary material for: Systematic review of patient-specific pre-operative predictors of pain improvement to endometriosis surgery
Source: Reprod Fertil. 2021 Mar 3;2(1):69–80. doi: 10.1530/RAF-20-0057 (PMC8812445; doi:10.1530/RAF-20-0057)
Supplement: Appendix S1 [file supplementary_material.pdf]

Appendix S1

Details of search strategy used in the systematic review on predictors of pain improvement after laparoscopic surgery for endometriosis

MEDLINE search strategy

1. (Validat* OR Predict* OR Rule*).ti,ab
2. (Predict* AND (Outcome* OR Risk* OR Model*)).ti,ab
3. ((Clinic* OR Presentation OR symptom* OR sign* OR History OR Variable* OR Criteria OR Scor* OR Characteristic* OR Finding* OR Factor*) AND (Predict* OR Model* OR Decision* OR Identif* OR Prognos* OR causality OR etiology OR odds ratio OR risk OR risk factor* OR odds OR cause)).ti,ab
4. (Decision* AND (Model* OR Clinical* OR Logistic Model*)).ti,ab
5. (Prognostic AND (History OR Variable* OR Criteria OR Scor* OR Characteristic* OR Finding* OR Factor* OR Model*)).ti,ab
6. ("risk score" OR "prediction model" OR "prediction rule" OR "risk assessment" OR "algorithm").ti,ab
7. (1 OR 2 OR 3 OR 4 OR 5 OR 6)
8. (endometrios*).all fields
9. (7 AND 8)
