# Supplementary material for: Systematic review of patient-specific pre-operative predictors of pain improvement to endometriosis surgery
Source: Reprod Fertil. 2021 Mar 3;2(1):69–80. doi: 10.1530/RAF-20-0057 (PMC8812445; doi:10.1530/RAF-20-0057)
Supplement: Table S1 Included and excluded studies [file supplementary_table_1.pdf]

Table S1
Included and excluded studies

Included studies	
Author	Title and reference
Abbott 2003	The effects and effectiveness of laparoscopic excision of endometriosis: a prospective study with 2-5 year follow-up. Hum Reprod. 2003;18(9):1922-7.
Chopin 2005	Chopin N, Vieira M, Borghese B, Foulot H, Dousset B, Coste J, et al. Operative management of deeply infiltrating endometriosis: results on pelvic pain symptoms according to a surgical classification. J Minim Invasive Gynecol. 2005;12(2):106-12.
Banerjee 2006	Banerjee S, Ballard KD, Lovell DP, Wright J. Deep and superficial endometriotic disease: the response to radical laparoscopic excision in the treatment of chronic pelvic pain. Gynecological Surgery. 2006;3(3):199-205.
Milingos 2006	Milingos S, Protopapas A, Kallipolitis G, Drakakis P, Loutradis D, Liapi A, et al. Endometriosis in patients with chronic pelvic pain: is staging predictive of the efficacy of laparoscopic surgery in pain relief? Gynecol Obstet Invest. 2006;62(1):48-54.
Ghai 2020	Ghai V, Jan H, Shakir F, Kent A. Identifying Preoperative Factors Associated with Nonresponders in Women Undergoing Comprehensive Surgical Treatment for Endometriosis. J Minim Invasive Gynecol. 2020;27(1):141-7.

Excluded studies	
Noventa M, Saccardi C, Litta P, et al. Ultrasound techniques in the diagnosis of deep pelvic endometriosis: algorithm based on a systematic review and meta-analysis. *Fertil Steril*. 2015;104(2):366-83	does not fit PICOS
Soriano D, Schonman R, Nadu A. et al. Multidisciplinary team approach to management of severe endometriosis affecting the ureter: long-term outcome data and treatment algorithm. J Minim Invasive Gynecol. 2011;18:483–488	no comparison group
Cocco A, Borghero A, Saccardi C, Guidetti G, Conte L, Litta P. Deep pelvic endometriosis: From diagnosis to wellness. Gynecological Surgery. 2009;6(SUPPL. 1):S39.	no comparison group, conference abstract
Daraï E, Dubernard G, Coutant C, Frey C, Rouzier R and Ballester M. Randomized trial of laparoscopically assisted versus open colorectal resection for endometriosis: morbidity, symptoms, quality of life, and fertility. Ann Surg 2010; 251:1018–1023.	does not fit PICOS
Daraï E, Thomassin I, Barranger E. et al. Feasibility and clinical outcome of laparoscopic colorectal resection for endometriosis. Am J Obstet Gynecol. 2005;192:394–400	does not fit PICOS
Deng S, Leng J, Lang J, Dai Y, Li X. [Clinicopathological characteristics of recurrent endometriosis and the outcomes of secondary surgery]. Zhonghua fu chan ke za zhi. 2011;46(11):809-12.	no comparison group
Dibi R, Pinho De Oliveira MA, Nogueira M, Muller M, Soares T, Souza C, et al. How many surgeries are necessary for definitively treatment of deep endometriosis. Journal of Minimally Invasive Gynecology. 2012;19(6 SUPPL. 1):S98.	does not fit PICOS, conference abstract
Dowaji J, Jaenicke F. Long-term results of laparoscopic resection of deep pelvic endometriosis with rectosigmoid involvement. Gynecological Surgery. 2010;7(SUPPL. 1):S160.	no comparison group

Dubernard G, Piketty M, Rouzier R, Houry S, Bazot M, Darai E. Quality of life after laparoscopic colorectal resection for endometriosis. Human reproduction (Oxford, England). 2006;21(5):1243-7.	no comparison group
Duffy JMN, Arambage K, Correa FJS, Olive D, Farquhar C, Garry R, et al. Laparoscopic surgery for endometriosis. The Cochrane database of systematic reviews. 2014 (4):CD011031.	not a trial
Grundstrom H, Alehagen S, Bertero C, Kjolhede P. Impact of Pelvic Pain and Endometriosis on Patient-Reported Outcomes and Experiences of Benign Hysterectomy: A Study from the Swedish National Register for Gynecological Surgery. Journal of Women's Health. 2018;27(5):691-8.	does not fit PICOS
Healey M, Ang C, Cheng C. Surgical treatment of endometriosis: a prospective randomized double-blind trial comparing excision and ablation. Fertil Steril 2010; 94: 2536–	does not fit PICOS
Ianieri MM, Mautone D, Ceccaroni M. Recurrence in Deep Infiltrating Endometriosis: A Systematic Review of the Literature. Journal of Minimally Invasive Gynecology. 2018;25(5):786-93.	does not fit PICOS
Jarrell J. Annual repeat rates of laparoscopic surgery: a marker of practice variation. American journal of medical quality : the official journal of the American College of Medical Quality. 2010;25(5):378-83.	does not fit PICOS
Kalu E, McAuley W, Richardson R. Teenagers, adolescents, endometriosis and recurrence: A retrospective analysis of recurrence following primary operative laparoscopy. Gynecological Surgery. 2008;5(3):209-12.	does not fit PICOS
Kayani S, Nightingale A. Laparoscopic surgery for endoemtriosis: A systematic review. Gynecological Surgery. 2009;6(SUPPL. 1):S85-S6.	does not fit PICOS
Li H-j, Leng J-h, Lang J-h, Wang H-l, Liu Z-f, Sun D-w, et al. [Correlative factors analysis of recurrence of endometriosis after conservative surgery]. Zhonghua fu chan ke za zhi. 2005;40(1):13-6.	does not fit PICOS
Martin CE, Johnson E, Wechter ME, Leserman J, Zolnoun DA. Catastrophizing: a predictor of persistent pain among women with endometriosis at 1 year. Human reproduction (Oxford, England). 2011;26(11):3078-84.	does not fit PICOS
Martin DC. Hysterectomy for treatment of pain associated with endometriosis. J Minim Invasive Gynecol 2006; 13:566–572.	does not fit PICOS
Meuleman C, Tomassetti C, D'Hooghe T M. Clinical outcome after laparoscopic radical excision of endometriosis and laparoscopic segmental bowel resection. Curr Opin Obstet Gynecol. 2012	does not fit PICOS
Moini A, Arabipoor A, Ashrafinia N. Risk factors for recurrence rate of ovarian endometriomas following a laparoscopic cystectomy. Minerva medica. 2014;105(4):295-301.	does not fit PICOS
Renner S P, Rix S, Boosz A. et al. Preoperative pain and recurrence risk in patients with peritoneal endometriosis. Gynecol Endocrinol. 2009 (2010 on file);28:1–6	does not fit PICOS
Saleh A, Tulandi T. Reoperation after laparoscopic treatment of endometriomas by excision and fenestration. Fertil Steril. 1999	does not fit PICOS
Sutton CJ, Ewen SP, Whitelaw N and Haines P. Prospective, randomized, double-blind, controlled trial of laser laparoscopy in the treatment of pelvic pain associated with minimal, mild, and moderate endometriosis. Fertil steril 1994; 62:696–700.	data not presented
Ford J, English J, Miles W A. et al. Pain, quality of life and complications following the radical resection of rectovaginal endometriosis. Br J Obstet Gynaecol. 2004;111:353–356.	no comparison group

Abbott J, Hawe J, Hunter D. et al. Laparoscopic excision of endometriosis: a randomized, placebo-controlled trial. Fertil Steril. 2004;82:878–884	no comparison group
Chapron C, Dubuisson JB. Laparoscopic treatment of deep endometriosis located on the uterosacral ligaments. Hum Reprod 1996;11:868–73.	no comparison group
Vercellini P, Fedele L, Aimi G, De Giorgi O, Consonni D and Crosignani PG. Reproductive performance, pain recurrence and disease relapse after conservative surgical treatment for endometriosis: the predictive value of the current classification system. Hum Reprod 2006a; 21:2679–2685	does not fit PICOS
Harris A, McCaughey T, Tsaltas J, Davies-Tuck M, Ratner R, Najjar H, et al. Endometriosis-related pelvic pain following laparoscopic surgical treatment J Endometr Pelvic Pain Disord [Internet]. 2020.	follow up too short
